# Cohort Profile: The Alliance for Maternal and Newborn Health Improvement (AMANHI) biobanking study

**DOI:** 10.1093/ije/dyab124

**Published:** 2021-08-24

**Authors:** Fahad Aftab, Salahuddin Ahmed, Said Mohammed Ali, Shaali Makame Ame, Rajiv Bahl, Abdullah H Baqui, Nabidul Haque Chowdhury, Saikat Deb, Usha Dhingra, Arup Dutta, Tarik Hasan, Aneeta Hotwani, Muhammad Ilyas, Mohammad Javaid, Fyezah Jehan, Mohamed Hamad Juma, Farah Khalid, Rasheda Khanam, Alexander Ansah Manu, Usma Mehmood, Nicole Minckas, Dipak Kumar Mitra, Imran Nisar, Ozren Polašek, Sayedur Rahman, Igor Rudan, Muhammad Sajid, Sunil Sazawal, Sachiyo Yoshida, the AMANHI biobanking study group

**Affiliations:** 1 Center for Public Health Kinetics, Global Zanzibar, Tanzania; 2 Projahnmo Research Foundation, Bangladesh; 3 Public Health Laboratory-IdC, Pemba, Zanzibar, Tanzania; 4 Department for Maternal, Newborn, Child, and Adolescent Health, and Ageing, World Health Organization, Geneva, Switzerland; 5 International Center for Maternal and Newborn Health, Department of International Health, Johns Hopkins Bloomberg School of Public Health, Baltimore, MD, USA; 6 Center for Public Health Kinetics, New Delhi, India; 7 The Department of Paediatrics and Child Health, Aga Khan University, Karachi, Sind Pakistan; 8 Department of Epidemiology and Disease Control, University of Ghana School of Public Health, Legon, Accra, Ghana; 9 North South University, Dhaka, Bangladesh; 10 University of Split School of Medicine, Split, Croatia; 11 Gen–info Ltd, Zagreb, Croatia; 12 Centre for Global Health, The Usher Institute, University of Edinburgh, Edinburgh, UK

Key Features

- The AMANHI Biobank cohort is a large cohort of pregnant women and their babies in sub-Saharan Africa and South Asia aimed at studying the interactions between genes and a wide range of varying environmental exposures on key pregnancy and birth outcomes.
- The cohort is well characterized for clinical, epidemiological and socio-economic information with harmonized data collection across all sites. The samples were collected and stored following standard operating procedures and provide an excellent opportunity for biological characterization.
- The cohort includes a total of 10 001 women enrolled between May 2014 and June 2018 across Sylhet-Bangladesh, Karachi-Pakistan and Pemba Island-Tanzania, who have given birth to 9938 babies.
- Follow-up included three to four visits during pregnancy: at baseline, at 24–28 weeks, at 32–36 weeks and after 37 completed weeks of gestational age to collect routine epidemiological data and biological samples, and two additional visits after birth: between 1 and 6 days after birth and the second one between 42 and 60 days of age, in which the newborn’s samples were taken.
- The data set comprises a wide range of phenotypical data and environmental measures, biological samples, as well as a multiplicity of outcomes from the mother, the fetus and the neonate.
- The AMANHI biobank data is available at the Department for Maternal, Newborn, Child and Adolescent Health, and Ageing at the World Health Organization, which is the coordination centre of the study. Queries regarding the data and potential collaborations can be sent to Dr Rajiv Bahl (bahlr@who.int).

## Why was the cohort set up?

Despite remarkable progress in the momentum generated by the millennium development goals, the levels of neonatal and maternal morbidity and mortality remain significantly high.^1^ Annually, an estimated 2.5 million neonatal deaths, 2.6 million stillbirths and 289 000 maternal deaths occur worldwide; south Asian and sub-Saharan African regions bear the highest burden.^2–4^ Many low- and middle-income countries (LMICs) experience a considerably diverging pattern of neonatal and maternal mortality, with more than half of the deaths directly attributable to pregnancy-associated complications such as pre-eclampsia, birth asphyxia, preterm births, intrauterine growth restriction (IUGR) or congenital anomalies.^5^ Although there are effective interventions to alleviate the consequences of these complications, there are few preventive strategies, with limited effectiveness. To address such a challenge, a better understanding of the biological mechanisms underlying the pathophysiology of adverse pregnancy outcomes and the identification of biomarkers to predict pregnant women at risk of adverse outcomes will allow women to be preferentially referred to hospitals for more complete work-up and management. This advancement in the care of women and infants will substantially reduce the rates of maternal, fetal and newborn morbidity and mortality.^5^^,^^6^

Since the reporting of the sequencing of the human genome, there has been a rise in the powerful high-throughput analysis of human genetic material that has uncovered an avalanche of genome-wide association studies.^7–9^ Despite unrivalled progress in biomedicine, the discoveries were made in high-income countries (HICs) and may not be entirely pertinent to other populations. Variances between countries can alter the risks, severity and pathophysiology of pregnancy-related adverse outcomes. Therefore, discovering and validating specific biomarkers in LMIC settings is fundamental to increasing their clinical usefulness, and permitting the early assessment, timely referral and ideal management of pregnancy-related adverse events.

Prior to recent initiatives such as the Human Heredity and Health in Africa (H3Africa) by the National Institute of Health, USA,^10^^,^^11^ only a few African countries had biobanks^12^^,^^13^ and there were almost none in South Asia. Despite the rapidly increasing availability and progressively decreasing costs of these technologies, there are still no biobanks available in Africa and South Asia with a focus on maternal, fetal and neonatal health.^14^ In an effort to reduce the inequity in international research, promote capacity building and infrastructure, and switch research priorities, there is a need for biobanks in LMICs to investigate the pathogenesis of maternal and child morbidity and mechanisms of host resistance to maximize the public health and clinical relevance of research activities.^15^^,^^16^

To this end, the Alliance for Maternal and Newborn Health Improvement (AMANHI) initiative was created with the primary goal of establishing well-characterized harmonized cohorts of pregnant women and their babies in sub-Saharan Africa and South Asia.^17^ A leading objective has been to broaden knowledge on key pregnancy and birth outcomes on a sustained research platform, prove the feasibility of the implementation of such initiatives in LMICs and enhance capacity around biobanking. In doing so, we hope to demonstrate the potential of biobanks for reliable research into the main risk factors, morbidity and mortality of the diseases relevant to LMICs. We utilize this opportunity to study interactions between genes and a wide range of varying environmental exposures in causing diseases of several dimensions in LMICs.

## Who is in the cohort and how often have they been followed up?

To construct the AMANHI biobank cohort, households in three sites (Sylhet-Bangladesh, Karachi-Pakistan and Pemba Island-Tanzania) had their geographical coordinates collected and linked to a database through a unique identification (ID) number to allow longitudinal linkages. Trained fieldworkers (FWs) or community health workers (CHWs), predominantly women, performed home visits every 2–3 months to all women of reproductive age in the study area to enquire about pregnancy. If a woman reported or suspected a pregnancy, FWs ascertained the gestational age using the date of her last menstrual bleeding and conducted a urine pregnancy test to confirm it. Pregnant women who provided consent underwent a screening ultrasound and Hadlock’s criteria were used to date the pregnancy more precisely.^18^ All women with early pregnancies between 8 and 19 weeks of gestation who intended to stay in the study areas for the entire duration of follow-up and consented for the collection of epidemiological data as well as biological samples were included in the cohort.

The AMANHI biobank cohort includes a total of 10 001 women across sites enrolled between from May 2014 and June 2018, who have given birth to 9938 babies and had their epidemiological data and biological samples collected and stored. The socio-demographic characteristics of the households and women enrolled in the cohort by site are presented in Table 1.

**Table 1:** Baseline socio-demographic characteristics of the households from women participating in the AMANHI (Alliance for Maternal and Newborn Health Improvement) biobank cohort

	Sylhet-Bangladesh	Pemba-Tanzania	Karachi-Pakistan	Total
Number of enrolled women (*N*)	3000	4501	2500	10 001
Woman's age in current pregnancy (years) [*n* (%)]				
15–19	634 (21.1%)	295 (6.55%)	197 (7.9%)	1126 (11.3%)
20–29	2004 (66.8%)	2429 (53.97%)	1594 (63.8%)	6027 (60.3%)
30–39	353 (11.8%)	1538 (34.17%)	681 (27.2%)	2572 (25.7%)
40–49	5 (0.3%)	239 (5.31%)	28 (1.1%)	272 (2.7%)
No data	4	0	0	4
Gravidity (including current pregnancy) [*n* (%)]				
1	988 (32.9%)	785 (17.5%)	478 (19.1%)	2251 (22.6%)
2	744 (24.8%)	441 (9.8%)	526 (21.0%)	1711 (17.1%)
3	544 (18.1%)	653 (14.6%)	471 (18.8%)	1668 (16.7%)
>4	720 (24.0%)	2606 (58.1%)	1025 (41.0%)	4351 (43.6%)
No data	4	16	0	20
Parity [*n* (%)]				
First pregnancy	988 (33.0%)	785 (17.5%)	478 (19.1%)	2251 (22.6%)
0	142 (4.7%)	71 (1.6%)	157 (6.3%)	370 (3.7%)
1–2	1294 (43.2%)	1163 (25.9%)	1092 (43.7%)	3549 (35.6%)
3–4	462 (15.4%)	1072 (23.9%)	475 (19.0%)	2009 (20.1%)
>5	110 (3.7%)	1394 (31.1%)	298 (11.9%)	1802 (18.1%)
No data	4	16	0	20
Woman’s education [*n* (%)]				
No schooling	190 (6.4%)	601 (13.4%)	1306 (52.2%)	2097 (21.0%)
1–4 years	278 (9.3%)	1499 (33.4%)	162 (6.5%)	1939 (19.4%)
5–9 years	2063 (69%)	2304 (51.4%)	597 (23.9%)	4964 (49.8%)
10+ years	460 (15.4%)	81 (1.8%)	435 (17.4%)	976 (9.8%)
No data	9	16	0	25
Woman's occupation [*n* (%)]				
Government/private job, daily wage earner	33 (1.1%)	581 (13.0%)	44 (1.8%)	658 (6.6%)
Self-employed	6 (0.2%)	261 (5.8%)	34 (1.4%)	301 (3.0%)
Farming	1 (0%)	778 (17.3%)	0 (0%)	779 (7.8%)
Not working outside home	2955 (98.7%)	2865 (63.9%)	2422 (96.9%)	8242 (82.6%)
No data	5	16	0	21
Father's education [*n* (%)]				
No schooling	485 (16.5%)	484 (10.8%)	1385 (55.4%)	2354 (23.7%)
1–4 years	594 (20.2%)	1386 (30.9%)	83 (3.3%)	2063 (20.8%)
5–9 years	1493 (50.8%)	2491 (55.5%)	566 (22.6%)	4550 (45.9%)
10+ years	367 (12.5%)	124 (2.8%)	466 (18.6%)	957 (9.6%)
No data	61	16	0	77
Number of persons per room in the house [median (IQR)]	1.7 (1.3-2.25)	1.5 (1-2)	3.5 (2.5-4.5)	2 (1.3-2.7)
Use biomass fuel for cooking [*n* (%)]	2933 (97.8%)	3799 (84.4%)	270 (10.8%)	7002 (70.0%)
Flush or pour toilet [*n* (%)]	1095 (36.6%)	1230 (27.4%)	2483 (99.3%)	4808 (48.1%)
Households with piped water into the dwelling [*n* (%)]	49 (1.6%)	1508 (33.6%)	1484 (59.4%)	3041 (30.4%)

IQR, interquartile range.

After enrolment, trained study FWs (or trained CHWs) conducted four home visits to all women in the cohort; at baseline (immediately after enrolment), at 24–28 weeks, 32–36 weeks and after 37 completed weeks of pregnancy to collect routine study data. During the pregnancy visits, maternal blood and urine samples were collected. Women were randomized for antenatal maternal sample collection at either 24–28 or 32–36 weeks’ gestation at a ratio of 2:1.

**Figure 1:**
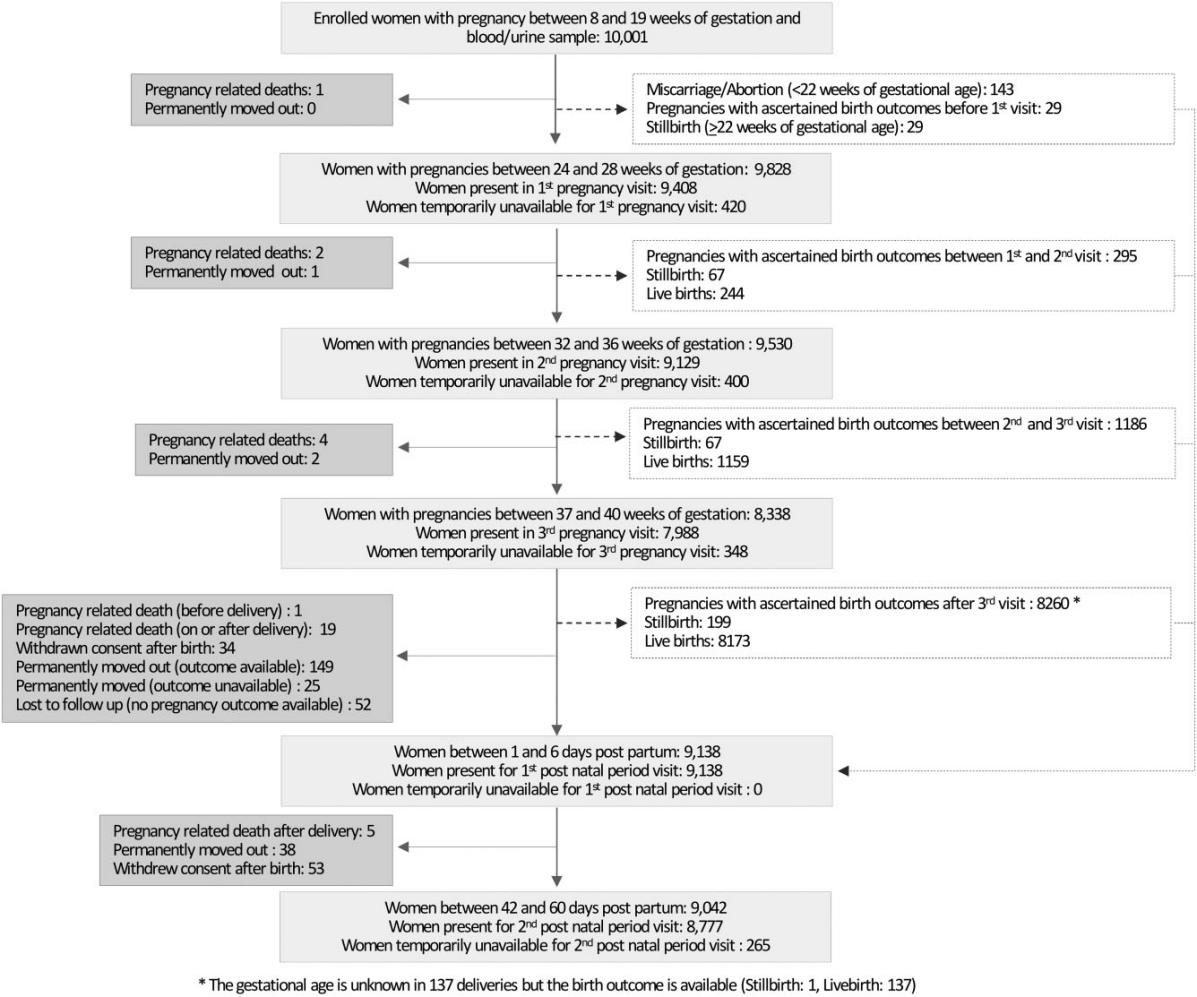
Flowchart of the inclusion route and follow-up for all eligible women.

Maternal stool, umbilical cord blood and tissue, placenta tissue and membranes, and infant saliva samples (where cord blood was not available) were obtained at birth and a delivery form was filled in within 48 hours of birth. Afterwards, two additional visits were made: one between 1 and 6 days after birth and the second one between 42 and 60 days of age, in which newborn’s blood and stool samples were taken. Figure 1 shows the flow of participants from enrolment to postnatal visits.

### Ethical considerations

FWs consented pregnant women, in their local or preferred languages, to undergo a screening ultrasound scan to date the pregnancies accurately. They enrolled women if the ultrasound-estimated gestational age of the pregnancy was within the eligibility cut-offs (gestation ≥8 to <20 weeks). Women were consented for the screening scan, follow-up and biosample collection. The fathers of the babies also consented for their saliva sample collection. These samples are only to be used for the goal of improving maternal and newborn health, and not for commercial purposes or for personal or institutional financial gain through the generation of intellectual property. A mechanism has been established to assure that data and specimens are shared in a manner that is consistent with the informed consent, that the participants’ privacy and confidentiality are protected, and that any future use of the data and specimens has Institutional Ethics Committee approval.

The study has received ethical approval from the local and institutional ethics committees of all the three sites: ICDDR, B and John Hopkins University for Bangladesh, Aga Khan University for Pakistan and ZAMREC and John Hopkins University for Tanzania. The protocols were also approved by the World Health Organization (WHO) Ethics Review Committee and continuing approvals were obtained each new year. There are no direct benefits of the study to the participants.

## What has been measured?

### Epidemiological, clinical and phenotypic data

As presented in Table 1 and to further characterize the women in the AMANHI cohort, information regarding maternal characteristics and medical history such as previous obstetric and gynaecological history, birth defects and congenital anomalies among previous babies, stillbirths and IUGRs, risk factors and exposures (cigarette smoking, alcohol ingestion, pregnancy physical work and the use of narcotics or other drugs) were collected at baseline and in the pregnancy visits.

During the pregnancy and postnatal visits, FWs gathered clinical data on the current pregnancy including maternal weight and height, morbidities and pregnancy complications, depression screening (nine-question patient health questionnaire) and exposure to risk factors such as smoking, alcohol ingestion, strenuous physical work and the use of narcotics and other drugs. A thorough nutritional assessment using a validated food-frequency questionnaire was carried out during one pregnancy visit.^19^ Urine dipstick examinations for proteinuria and blood-pressure recordings using a Microlife^®^ WatchBP^®^ Home monitor were undertaken at each visit. Table 2 summarizes the main medical history and clinical characteristics of the AMANHI cohort.

**Table 2:** Summary of the obstetric history and current risk factors of women participating in the AMANHI (Alliance for Maternal and Newborn Health Improvement) biobank cohort

	Sylhet-Bangladesh	Pemba-Tanzania	Karachi-Pakistan	Total
Number of women with a previous pregnancy (*N*)	2008	3716	2022	7746
Age at first pregnancy [median (IQR)]	18 (18-20)	19 (17-21)	19 (17-22)	19 (18-21)
ANC in previous pregnancies:				
Attended ANC in all previous pregnancies	671 (33.4%)	3379 (91.3%)	1216 (60.1%)	5266 (68.1%)
Attended ANC in some of the previous pregnancies	788 (39.2%)	311 (8.4%)	636 (31.5%)	1735 (22.5%)
No ANC in the previous pregnancies	549 (27.3%)	10 (0.3%)	170 (8.4%)	729 (9.4%)
No data	0	16	0	16
History of miscarriage	382 (19.0%)	805 (21.7%)	746 (36.9%)	1933 (25.0%)
Number of women with a previous pregnancy that finished in a birth (*N*)	1931	3716	1904	7461
History of stillbirth	235 (12.2%)	360 (9.7%)	160 (8.4%)	755 (10.1%)
History of caesarean sections	62 (3.2%)	118 (3.2%)	192 (10.1%)	372 (5.0%)
History of severe bleeding or haemorrhage before delivery	14 (0.7%)	141 (3.8%)	41 (2.2%)	196 (2.6%)
History of severe bleeding or haemorrhage after delivery	22 (1.1%)	253 (6.8%)	41 (2.2%)	316 (4.2%)
History of premature birth	108 (5.6%)	112 (3.0%)	177 (9.3%)	397 (5.3%)
History of multiple pregnancies (twins or triplets)	28 (1.5%)	119 (3.2%)	26 (1.4%)	173 (2.3%)
History of pre-eclampsia/eclampsia	3 (0.2%)	53 (1.4%)	37 (1.9%)	93 (1.2%)
Women who have had any babies with birth defects	25 (1.3%)	137 (3.7%)	54 (2.9%)	216 (2.9%)
Women with self-reported illness before current pregnancy (*N*)	3000	4501	2500	10 001
Diabetes	10 (0.3%)	25 (0.6%)	19 (0.8%)	54 (0.5%)
Thyroid disease	3 (0.1%)	11 (0.3%)	12 (0.5%)	26 (0.3%)
Cardiac disease	4 (0.1%)	22 (0.5%)	7 (0.3%)	33 (0.3%)
Epilepsy	11 (0.4%)	24 (0.6%)	2 (0.1%)	37 (0.4%)
Hypertension	7 (0.2%)	130 (3.1%)	143 (5.7%)	280 (2.8%)
HIV/AIDS	0 (0%)	7 (0.2%)	3 (0.1%)	10 (0.1%)
Tuberculosis	6 (0.2%)	18 (0.4%)	32 (1.3%)	56 (0.6%)
Woman's risk factors and exposures during current pregnancy (*N*)	3000	4501	2500	10 001
Woman's BMI (kg/m^2^)				
Underweight (<18.5)	964 (32.5%)	251 (6.3%)	518 (22.1%)	1733 (18.6%)
Normal weight (18.5–24.9)	1807 (60.9%)	2149 (53.6%)	1208 (51.6%)	5164 (55.4%)
Overweight (25.0–29.9)	165 (5.6%)	960 (23.9%)	439 (18.8%)	1564 (16.8%)
Obesity (>30.0)	31 (1.0%)	652 (16.2%)	175 (7.5%)	858 (9.2%)
No data	33	489	160	682
Woman's height (cm) [mean (SD)]	149.7 (5.4)	155.3 (6.3)	153.7 (6.5)	153.1 (6.5)
Ever use tobacco (smoke or sniffed/chewed tobacco)	511 (17.0%)	32 (0.8%)	503 (20.1%)	1046 (10.5%)
Second-hand smoking^a^	2365 (78.9%)	1054 (23.4%)	727 (29.1%)	4146 (41.5%)
Ever consume alcohol	0 (0.0)	3 (0.1%)	32 (1.3%)	35 (0.3%)

a We defined second-hand smoking as having lived with a smoker in the same room or compound.

ANC, antenatal care; BMI, body mass index; IQR, interquartile range; SD, standard deviation.

### Biomaterial collection and biobanking

A sampling scheme with a sequence of time points was used to obtain maternal blood and urine, maternal stool, umbilical cord blood and tissue, placenta tissue and membranes, newborn stool and saliva samples (where cord blood was not available) and paternal saliva samples. Standardized protocols for collection, processing and storage were implemented across all sites, as shown in Supplementary Table S1, available as Supplementary data at *IJE* online, and previously presented in the methodological paper.^17^ The biological samples are stored separately in the AMANHI biobank at each site. In Bangladesh, the AMANHI biobank is located in Sylhet and in the North South University, Dhaka. In Pakistan, the AMANHI biobank is at The Aga Khan University, Stadium Campus Karachi. In Pemba, it is in CPHK-PHL-IdC biorepository situated at Public Health Laboratory-Ivo de Carneri (PHL-IdC) Campus, Wawi, Chake district of Pemba Island Zanzibar.

During the enrolment visit, blood was drawn for all participating women and a urine sample was collected (Table 3). The blood was collected into pre-labelled tubes, centrifuged and serum, plasma and buffy-coat aliquots were obtained. Maternal urine samples (*n* = 10 001) were similarly centrifuged and RNALater was mixed with sediments and aliquots taken for storage. Maternal blood and urine samples were taken at either the 24–28 or 32–36 weeks’ (blood samples: *n* = 9134; urine samples: *n* = 9141) pregnancy visit and after 42 days of the delivery (blood samples: *n* = 8743; urine samples: *n* = 8746).

**Table 3:** Numbers of blood, urine, saliva, stool and placental samples per study site

		Sylhet-Bangladesh	Pemba-Tanzania	Karachi-Pakistan	Total
Sample type	Sample time	*n*/*N* (%)	*n*/*N* (%)	*n*/*N* (%)	*n*/*N* (%)
Maternal blood^a^	Enrolment (8 to <20 weeks)	2999/3000 (100%)	4501/4501 (100%)	2500/2500 (100%)	10000/10001 (100%)
	Pregnancy visit 2 or pregnancy visit 3	2760/2958 (93.3%)	4170/4423 (94.3%)	2204/2427 (90.8%)	9134/9808 (93.1%)
	Visit during postnatal period (42 days post-partum)	2529/2731 (92.6%)	4007/4473 (89.6%)	2207/2330 (94.7%)	8743/9534 (91.7%)
Maternal urine^a^	Enrolment (8 to <20 weeks)	3000/3000 (100%)	4501/4501 (100%)	2500/2500 (100%)	10 001/10 001 (100%)
	Pregnancy visit 2 or pregnancy visit 3	2767/2958 (93.5%)	4170/4423 (94.3%)	2204/2427 (90.8%)	9141/9808 (93.2%)
	Visit during postnatal period (42 days post-partum)	2534/2731 (92.8%)	4007/4473 (89.6%)	2205/2330 (94.6%)	8746/9534 (91.7%)
Faeces^b^	Maternal	2581/3000 (86%)	3869/4440 (86%)	2201/2287 (96.2%)	8651/9727 (88.9%)
	Newborn	2213/2858 (77.4%)	3585/4366 (82.1%)	1999/2205 (90.7%)	7797/9429 (82.7%)
Placenta^c^	Placenta	2532/2964 (85.4%)	4095/4443 (92.2%)	808/2325 (34.8%)	7435/9732 (76.4%)
Cord blood^c^	Fresh cord blood	1488/2964 (50.2%)	3674/4443 (82.7%)	294/2325 (12.6%)	5456/9732 (56.1%)
	Clotted cord blood^e^	567/2964 (19.1%)	377/4443 (8.5%)	517/2325 (22.2%)	1461/9732 (15.0%)
Saliva	Newborn^d^	553/2858 (19.3%)	206/4366 (4.7%)	1413/2325 (60.8%)	2172/9549 (22.7%)
	Paternal	2384/3000 (79.5%)	3858/4501 (85.7%)	2319/2500 (92.8%)	8561/10 001 (85.6%)

a To calculate the proportion of maternal blood and urine samples collected during the pregnancy visits, the denominator is the total number of women still pregnant in the cohort at the time of the visit. For the maternal samples collected in the postnatal period, the denominator corresponds to the total number of women who had a stillbirth or live birth.

b To calculate the proportion of newborn faeces collected, we included all babies born alive in the denominator.

c To calculate the proportion of placenta and cord blood samples collected, we considered all pregnancies that ended in delivery as the denominator.

d Collected only if cord blood could not be collected.

e Collected only if fresh cord blood could not be collected.

At birth, 7435 placenta samples were collected, and 5456 fresh and 1461 clotted cord blood samples extracted. Placenta samples were harvested and processed within 30 minutes of delivery. Full-thickness tissue samples were harvested in four areas, three of which have a thin layer of maternal tissues sliced off the surface. Placental tissue samples were stored in RNALater, Formalin, and were flash-frozen.

A total of 8651 maternal stool samples were collected to assess the microbiota at the time of delivery and 7797 newborn stool samples were collected when feeding started. A single sample of paternal saliva (during one of the pregnancy visits or during the postnatal period) and newborn saliva (between 42 and 60 days post-partum from babies whose cord blood could not be obtained at the time of birth) were collected using an Oragene DNA collection kit for DNA extraction. All biological samples were processed and stored at –80°C.

## What has been found?

So far, data from the AMANHI biobank study have been analysed to examine socio-demographic characteristics, obstetric history, maternal morbidities and birth outcomes. We identified 10 001 pregnancies across the study sites, with outcomes ascertained for 9921 (99.2%), including 143 (1.4%) miscarriages or induced abortions occurring after enrolment (from 8 to <22 weeks of gestational age) and 8 pregnancy-related death occurring before delivery. The remaining 9850 pregnancies resulted in a total of 9938 births, with 9576 live-born babies and 362 stillbirths (4%). There was a total of 32 pregnancy-related deaths (deaths during pregnancy, childbirth and within 42 days post-partum).

The proportion of maternal morbidities varied widely across study sites, ranging from 9% of women with at least one pregnancy-related morbidity in Bangladesh to 41% in Karachi. This variation was mainly driven by the high incidence of infections in the antepartum and post-partum periods in Karachi (23% and 13%, respectively) in comparison with the other two sites. Pregnancy-related hypertension was the most common cause of maternal morbidity in Pemba and Karachi. Substantial differences were found in the burden of pre-eclampsia/eclampsia when analysing by region, being over 6-fold higher in the African site in comparison with the Asian sites. Table 4 presents the distribution of these morbidities by site. Of the 1517 women with some morbidity, 76% (*n* = 1146) had only one whereas the remaining 24% (*n* = 371) had two or more.

**Table 4:** Pregnancy characteristics and women’s morbidities during current pregnancy

	Sylhet-Bangladesh	Pemba-Tanzania	Karachi- Pakistan	Total
Current pregnancy and delivery characteristics (*N*)	3000	4501	2500	10 001
Women's age at time of pregnancy [mean (SD)]	23.5 (4.4)	27.9 (6.3)	26.6 (5.2)	26.3 (5.8)
Multiple pregnancies [*n* (%)]	27 (0.9%)	90 (2.0%)	20 (0.8%)	137 (1.4%)
Pregnancies that ended in delivery (excluding miscarriages and PRD before delivery) (*N*)	2958	4415	2426	9799
Place of delivery [*n* (%)]^a^				
Facility/hospital	2035 (68.9%)	3549 (83.0%)	1568 (67.8%)	7152 (74.9%)
Home/compound	891 (30.2%)	720 (16.8%)	725 (31.3%)	2336 (24.5%)
Other	29 (1.0%)	8 (0.2%)	21 (0.9%)	58 (0.6%)
Missing	0	138	112	250
Delivery mode [*n* (%)]^a^				
Vaginal	2510 (85.3%)	4099 (95.8)	1856 (80.1%)	8465 (88.7%)
Vaginal assisted (e.g. forceps, vacuum)	18 (0.6%)	10 (0.2%)	63 (2.7%)	91 (1.0%)
Caesarean section	415 (14.1%)	168 (3.9%)	399 (17.2%)	982 (10.3%)
Missing	12	138	108	258
Person assisting the delivery [*n* (%)]^a^				
Doctor	354 (12.0%)	314 (7.3%)	1129 (49.4%)	1797 (18.9%)
Midwife/nurse	1671 (56.8%)	2915 (68.2%)	551 (24.1%)	5137 (54.0%)
Traditional birth attendant	767 (26.1%)	644 (15.1%)	576 (25.2%)	1987 (20.9%)
Other health professional	78 (2.7%)	397 (9.3%)	0 (0%)	475 (5.0%)
Relative/friend	58 (2.0%)	7 (0.2%)	25 (1.1%)	90 (0.9%)
None, woman herself	15 (0.5%)	0 (0%)	5 (0.2%)	20 (0.2%)
Missing	12	138	140	290
Women morbidity during current pregnancy (*N*)	3000	4501	2500	10 001
Severe antepartum haemorrhage [*n* (%)]	21 (0.7%)	61 (1.4%)	145 (5.9%)	227 (2.3%)
Late antepartum infection [*n* (%)]	52 (1.9%)	21 (0.5%)	532 (22.8%)	605 (6.0%)
Hypertensive disorders of pregnancy [*n* (%)]				
Gestational hypertension	104 (3.5%)	603 (13.7%)	243 (10%)	950 (9.5%)
Pre-eclampsia	5 (0.2%)	175 (3.9%)	15 (0.6%)	195 (1.9%)
Eclampsia	1 (0%)	4 (0.1%)	0 (0%)	5 (0.0%)
Post-partum haemorrhage [*n* (%)]	14 (0.5%)	35 (0.9%)	33 (1.4%)	82 (0.8%)
Post-partum infection [*n* (%)]	94 (3.4%)	141 (3.5%)	298 (12.8%)	533 (5.3%)

a Denominators include all pregnancies that ended in the delivery of either a stillbirth or a live birth.

PRD, pregnancy-related deaths; SD, standard deviation.

Karachi experienced the highest number of post-enrolment miscarriages with 20 miscarriages per 1000 pregnancies in comparison with 12 and 13 per 1000 pregnancies in Bangladesh and Tanzania, respectively. On the contrary, the number of pregnancy-related deaths (PRDs) in Karachi was 240 per 100 000 pregnancies, representing two-thirds of the PRDs in Pemba (378 per 100 000 pregnancies). Of all PRDs, 25% occurred before labour and the remaining 75% during labour, between birth and 24 hours, or within 42 days post-partum.

From all births, 9576 resulted in a live baby (96%), with clear variations in the neonatal outcomes across regions. Those babies born in Bangladesh and Pakistan were born earlier and with a lower weight than the babies born in Pemba. The incidence of prematurity (<37 weeks of gestational age) was, on average, double in Asia (15%) compared with that in Africa (7%). The same relation was present for birthweight, with 27% of live babies born weighing <2500 g in Asia vs 7% in Africa. When analysing the relationship between birth and time of gestation, Bangladesh had the highest proportion of small-for-gestational-age babies (SGA: 46%).

Overall, the proportion of premature births due to premature ruptures of the membranes (PPROM) was 20% of all preterm births, 75% from spontaneous preterm labour and only 5% because of a medically indicated caesarean section.

Neonatal deaths varied widely per region, with 47 neonatal deaths per 1000 live births in Bangladesh, 39 per 1000 live births in Pakistan and 20 per 1000 live births in Tanzania. Site-specific pregnancy and neonatal outcomes are shown in Table 5.

**Table 5:** Pregnancy and birth outcomes per study site

		Sylhet-Bangladesh	Pemba-Tanzania	Karachi- Pakistan	Total
Pregnancy outcomes	PRD [*n*/*N* (rate per 100 000 pregnancies)]	9/3000 (300)	17/4501 (378)	6/2500 (240)	32/10 001 (320)
	Miscarriage [*n*/*N* (rate per 1000 pregnancies)]^a^	36/3000 (12)	58/4501 (13)	49/2500 (20)	143/10 001 (14)
	Stillbirths [*n*/*N* (rate per 1000 births)]	127/2985 (43)	141/4507 (31)	94/2446 (38)	362/9938 (36)
	Live births [*n*/*N* (rate per 1000 births)]	2858/2985 (957)	4366/4507 (969)	2352/2446 (962)	9576/9938 (964)
Gestational age at birth among live births	Early preterm, <34 weeks [*n* (%)]	87 (3.0%)	81 (1.9%)	72 (3.1%)	240 (2.5%)
	Late Preterm, 34–36 weeks [*n* (%)]	276 (9.7%)	224 (5.3%)	312 (13.3%)	812 (8.6%)
	Term, ≥37 weeks [*n* (%)]	2495 (87.3%)	3954 (92.8%)	1967 (83.7%)	8416 (88.9%)
	No data on gestational age	0	107	1	108
Birthweight among live births	Low birthweight (<2500 g) [*n* (%)]	713 (30.1%)	261 (6.5%)	497 (23.4%)	1471 (17.3%)
	Normal birthweight (≥2500 g) [*n* (%)]	1656 (69.9%)	3773 (93.5%)	1624 (76.6%)	7053 (82.7%)
	No data on birthweight	489	332	231	1052
Birthweight-for-gestational age at birth among live birth	Small for gestational age [*n* (%)]	1089 (46.0%)	408 (10.2%)	690 (33.2%)	2187 (25.8%)
	Appropriate for gestational age [*n* (%)]	1289 (52.7%)	3083 (77.3%)	1319 (63.5%)	5691 (67.0%)
	Large for gestational age [*n* (%)]	31 (1.3%)	511 (12.8%)	69 (3.3%)	611 (7.2%)
	No data	489	361	274	1124
Deaths in the cohort^b^	Early neonatal deaths [*n* (rate per 1000 live births)]	113 (40)	77 (18)	77 (33)	267 (28)
	Late neonatal death [*n* (rate per 1000 live births)]	21 (7)	10 (2)	14 (6)	45 (5)

a The cut-off point for miscarriage/abortion was 22 weeks of gestational age.

b Early neonatal deaths include those deaths that occurred between days 0 and 6 of life, whereas late neonatal death occurred between days 7 and 28.

PRD, pregnancy-related deaths.

## What are the main strengths and weaknesses?

The AMANHI biobank is the first population-based repository of biological samples established in sub-Saharan Africa and South Asia—two regions that bear a substantial portion of the total global burden of maternal deaths, stillbirths and neonatal deaths. During this study, we collected extensive data on socio-economic and household characteristics, clinical and obstetric history, and maternal morbidities and phenotypical data by systematic and prospective follow-up. In addition, we documented a multiplicity of outcomes from the mother, the fetus and the neonate, opening opportunities to explore an array of new hypotheses. The high follow-up rates allowed the collection of large number of samples from pregnancy to delivery up to 42 days post-partum. The extraction of these samples followed a harmonized process, including the use of uniform protocols; the centralized procurement of equipment, materials and reagents; and the strict and rigorous quality-control measures that allow the comparability of data across all sites. To ensure a thorough process and reduce transcription errors, samples have been time-stamped and barcoded by a computer, and all metadata are available for analysis. The AMANHI biobank can be used as a platform for further exploration of new hypotheses and technologies, and to build local capacity in these low-resource settings for high-quality research and ultra-high-throughput analyses.

Although we had a limited number of pregnancies per site, we were able to collect harmonized samples from 10 001 pregnant women and their offspring, which will allow pooling and consequent use in the analyses of less frequent outcomes. Despite taking all methodological measures to collect the most adequate samples to conduct analyses of data from whole-genome arrays, not all analyses will be possible, as specific assays might have requirements that were not considered during the preparation of this study. Moreover, unlike biobanks in many HICs, we had to reduce the volume of blood per sample, which will affect the final number of aliquots available for analysis. Finally, despite having a well-characterized population-based cohort in these LMICs, it does not inform on the biological mechanisms underlying childhood linear growth and neurodevelopment. A continuation of the cohort following up the children into their second and third years after birth is now ongoing (All Children Thrive- ACT- study) and will be published separately. The ACT study will provide opportunities to examine epigenetic factors in pregnancy or early childhood that could predict stunting and impaired neurodevelopment—the origins and risk factors of susceptibility to infectious agents and non-communicable diseases.

## Can I get hold of the data? Where can I find out more?

Establishing this biobank is only the first step and will be of little value if it is not utilized to address current global challenges. The AMANHI biobank data are available at the Department for Maternal, Newborn, Child, and Adolescents Health, and Ageing at the WHO, which is the coordination centre of the study. Queries regarding the data and potential collaborations can be sent to Dr Rajiv Bahl (bahlr@who.int). To access the data, a formal application must be submitted with a detailed research proposal consisting of the proposed title, authors, affiliations, research question, brief scientific background of ∼200 words, study design, study population with details on eligible populations and exclusions, outcome and exposure variables, covariables and proposed statistical analysis. The AMANHI group will scrutinize the application. An AMANHI biobank sample utilization committee at each site will provide oversight for the access of data.

## Supplementary data

Supplementary data are available at *IJE* online.

## Ethics approval

The study has received ethical approval from ICDDR, B and John Hopkins University for Bangladesh, Aga Khan University for Pakistan and ZAMREC and John Hopkins University for Tanzania. The protocols were also approved by the WHO Ethics Review Committee (Approval number: RPC532).

## Funding

This work was supported by the Bill & Melinda Gates Foundation through a grant to the World Health Organization [Grant Number I64438]. The funders have played no role in the drafting of the manuscript and the decision to submit for publication.

## Data availability

The data underlying this article will be shared on reasonable request to the corresponding author.

## Supplementary Material

dyab124_Supplementary_Data

## Notes

All authors contributed equally to the paper and have been listed in alphabetical order.
