# Supplementary material for: Cohort Profile: The Alliance for Maternal and Newborn Health Improvement (AMANHI) biobanking study
Source: Int J Epidemiol. 2021 Aug 24;50(6):1780–1781i. doi: 10.1093/ije/dyab124 (PMC8743110; doi:10.1093/ije/dyab124)
Supplement: dyab124_Supplementary_Data [file dyab124_supplementary_data.zip › ije-2020-11-2176-File008.docx]

**Authors’ contributions**

Conceptualization, design and data interpretation: Said Mohammed Ali, Rajiv Bahl, Abdullah H. Baqui, Fyezah Jehan, Rasheda Khanam, Alexander Ansah Manu, Imran Nisar, Ozren Polašek , Igor Rudan, Sunil Sazawal.

Data acquisition, analysis and interpretation: Fahad Aftab, Salahuddin Ahmed, Shaali Makame Ame, Nabidul Haque Chowdhury, Saikat Deb, Usha Dhingra, Arup Dutta, Tarik Hasan, Aneeta Hotwani, Muhammad Ilyas, Mohammad Javaid, Mohammed Hamad Juma, Farah Khalid, Usma Mehmood, Nicole Minckas, Dipak Kumar Mitra, Sayedur Rahman, Muhammad Sajid, Sachiyo Yoshida.

Drafting and revising the manuscript: all authors listed above contributed to preparing, drafting and revising the manuscript, gave final approval of the published version and agreed to be accountable for all aspects of the work.
