# Supplementary material for: Cohort Profile: The Alliance for Maternal and Newborn Health Improvement (AMANHI) biobanking study
Source: Int J Epidemiol. 2021 Aug 24;50(6):1780–1781i. doi: 10.1093/ije/dyab124 (PMC8743110; doi:10.1093/ije/dyab124)
Supplement: dyab124_Supplementary_Data [file dyab124_supplementary_data.zip › ije-2020-11-2176-File009.docx]

Supplementary table. Type of available aliquots for analysis

| **Sample type** | **Aliquots** | **Timing of collection** |
| --- | --- | --- |
| **Maternal blood** | Whole blood samples | Enrolment, 24–28 weeks or 32–36 weeks, postnatal day 42–60 |
|  | Serum aliquots |  |
|  | Plasma aliquots |  |
|  | Buffy coat aliquots |  |
|  | Blood spots in Whatman cards |  |
| **Maternal urine** | Uncentrifugated urine samples | Enrolment, 24–28 weeks or 32–36 weeks, postnatal day 42–60 |
|  | Sediment aliquots stabilized with RNALater |  |
| **Faeces** | Maternal stool sample | At birth |
|  | Newborn stool sample | At birth |
| **Saliva** | Paternal saliva sample stored in Oragene DNA kit | Antenatal or postnatal |
|  | Newborn sample stored in Oragene DNA kit | Postnatal day 42–60 |
| **Placenta** | Tissue samples stored in RNALater, alcohol, or are flash frozen | At birth |
|  | Membranes samples stored in RNALater, alcohol, or are flash frozen |  |
| **Cord blood** | Blood spots in Whatman cards |  |
|  | Tissue samples in RNALater, alcohol, or formalin solution |  |
